# The True Inwardness of State Registration

**Published:** 1919-12-27

**Authors:** 


### THE TRUE INWARDNESS OF STATE REGISTRATION.

*To the Editor of* The Hospital.

Sir,—I have received the following letter from Lord Knutsford:—

Kneesworth Hall, Royston, Herts.
December 12, 1919.

To Ierne.

With reference to your article on State Registration, you rightly say that the nurses of Bart's, Guy's, or St. Thomas's—and you might have added the London and all other good training-schools—will gain nothing in status by describing themselves as R.N., and I agree entirely with your view that the only nurses who will benefit are "the birds of doubtful plumage," as you write. But then you go on to say that the State Registration of Nurses is absolutely essential to the Minister of Health, and that, with a view of his being able to measure the nursing strength of the country, the State Register will supply him with complete information, and that it will enable him to put his finger on the name, address, and qualification of every working nurse in the country. Will it? I think not. It would do so if no woman was allowed to nurse unless she were registered. But so long as registration is voluntary, and very especially if the good nurses realise that you have pointed out that they are to be herded with the "birds of doubtful plumage," Registration will not give the Minister anything of the sort.

It would be unthinkable that no woman shall be allowed to nurse unless she had the qualifications of a fully-trained nurse. There are thousands of places open for nurses with less training. But if instead of a Register of fully-trained nurses there was an official Directory of Nurses, it would be possible to make it penal to nurse for gain unless a woman had entered her name in that Directory. The Directory would contain the names of every woman nursing for gain, and would state exactly what her training had been—six months or six years.

By this means, and this only, would the Minister of Health get a measure of the nursing strength of the country. I do not know—do you?—of any useful voluntary registration? The dental profession has one, with the result that the registered dentists have been swamped by the unregistered, and this has become such a scandal that the Government appointed a Committee to report on the matter, and our report has just gone in. If our recommendations are carried out, no one will be allowed to practise unless he be registered. This, as I have said, is undesirable and impossible for the nursing profession.—Yours, Knutsford.

Lord Knutsford is Right.

This thoughtful people will admit. Before I received his letter I had realised the force of his argument, for I was informed by a Metropolitan nurse that she had no intention of registering. She regards her diploma as a superior qualification.

It may be within your recollection that some months ago I quoted the American system of organising an army of "practical" nurses as a solution of the difficulty. In America a "practical" nurse is a woman whose professional qualifications are of a lower standard than those of the trained nurse, who is willing to work at a lower scale of pay, and who will fulfil other household functions.

If Lord Knutsford's idea could be materialised, and nursing for gain confined to registered persons, properly graded, the economic position of the nurse, no matter what her professional status might be, would automatically rise.

I am bound to say, and I say it with pleasure, that Lord Knutsford's suggestion is a valuable contribution towards the solution of the nursing problem.

Trained nurses are now reaping the fruit of Mrs. Bedford Fenwick's teaching. She lured them in quest of a will o' the wisp. I wonder how many of them will find in their "R.N." qualification a means of increasing their expenditure! IERNE.

